# Potent synergy and sustained bactericidal activity of polymyxins combined with Gram-positive only class of antibiotics versus four Gram-negative bacteria

**DOI:** 10.1186/s12941-024-00720-4

**Published:** 2024-07-04

**Authors:** Yan Wang, Jianwen Feng, Jiameng Yu, Lirong Wen, Lidan Chen, Huijie An, Weibin Xiao, Bing Zhang, Huanhuan Feng, Mou Zhou, Zhihui Jiang

**Affiliations:** 1 School of Pharmaceutical Sciences, Southern Medical University, Guangzhou, 510515 China; 2 Department of Pharmacy, General Hospital of Southern Theatre Command, Guangzhou, 510010 China; 3 Graduate School, Guangzhou University of Chinese Medicine, Guangzhou, 510006 China; 4 School of Pharmaceutical Sciences, Dali University, Dali, 671003 China; 5 Department of Laboratory Medicine, General Hospital of Southern Theatre Command, Guangzhou, 510010 China; 6 Department of Clinical Pharmacy, General Hospital of Southern Theatre Command, Guangzhou, 510010 China; 7 Department of Healthcare, General Hospital of Southern Theatre Command, Guangzhou, 510010 China; 8 Department of Blood Transfusion Medicine, General Hospital of Southern Theatre Command, Guangzhou, 510010 China

**Keywords:** Polymyxins, Gram-negative bacteria, Oritavancin, Synergy, Time-kill assay

## Abstract

**Background:**

Gram-negative bacteria (GNB) are becoming increasingly resistant to a wide variety of antibiotics. There are currently limited treatments for GNB, and the combination of antibiotics with complementary mechanisms has been reported to be a feasible strategy for treating GNB infection. The inability to cross the GNB outer membrane (OM) is an important reason that a broad spectrum of Gram-positive only class of antibiotics (GPOAs) is lacking. Polymyxins may help GPOAs to permeate by disrupting OM of GNB.

**Objective:**

To identify what kind of GPOAs can be aided to broaden their anti-GNB spectrum by polymyxins, we systematically investigated the synergy of eight GPOAs in combination with colistin (COL) and polymyxin B (PMB) against GNB in vitro.

**Methods:**

The synergistic effect of COL or PMB and GPOAs combinations against GNB reference strains and clinical isolates were determined by checkerboard tests. The killing kinetics of the combinations were assessed using time-kill assays.

**Results:**

In the checkerboard tests, polymyxins-GPOAs combinations exert synergistic effects characterized by species and strain specificity. The synergistic interactions on *P. aeruginosa* strains are significantly lower than those on strains of *A. baumannii*, *K. pneumoniae* and *E. coli*. Among all the combinations, COL has shown the best synergistic effect in combination with dalbavancin (DAL) or oritavancin (ORI) versus almost all of the strains tested, with FICIs from 0.16 to 0.50 and 0.13 to < 0.28, respectively. In addition, the time-kill assays demonstrated that COL/DAL and COL/ORI had sustained bactericidal activity.

**Conclusions:**

Our results indicated that polymyxins could help GPOAs to permeate the OM of specific GNB, thus showed synergistic effects and bactericidal effects in the in vitro assays. In vivo combination studies should be further conducted to validate the results of this study.

**Supplementary Information:**

The online version contains supplementary material available at 10.1186/s12941-024-00720-4.

## Introduction

Antimicrobial resistance is a growing problem and one of the most serious threats facing humanity today. At the same time, GNB pathogens hold a significant position in the list of priority pathogens for antibiotic resistance published by the World Health Organization (WHO) in 2017 [1]. In hospitals, the number of antibiotics to treat Gram-negative resistant strains is becoming increasingly scarce and limited due to the emergence of multidrug-resistant (MDR), extensively-resistant (XDR) and pandrug-resistant (PDR) isolates. Polymyxins were developed in the 1940s, and the clinically marketed products are mainly PMB and COL sulfate and mesylate [2]. As cationic lipopeptide antibiotics, polymyxins have electrostatic interactions with negatively charged lipid A on lipopolysaccharide (LPS), leading to depolarization and disruption of the GNB outer membrane (OM), followed by cell death through osmotic lysis and the production of toxic hydroxyl radicals [3]. However, the intravenous formulations of COL and PMB were gradually abandoned by the early 1980s due to the reported high incidence of nephrotoxicity and the availability of alternative antibiotics with better safety profiles. Since it is difficult to develop new classes of antibiotics, polymyxins are reconsidered the last alternative for the treatment of GNB [4]. However, polymyxins monotherapy should be avoided in most cases [5]. The use of polymyxins combination therapy could be a favorable approach for decreasing the required dosage of polymyxins and ultimately leading to a reduction in nephrotoxicity.

The OM of GNB is the main cause of inactivity to a host of GPOAs [6]. Although GPOAs are not active against GNB alone, their activity is likely to be improved if used in combination with OM disruption agents [7–9]. GPOAs, mainly including glycopeptides class [vancomycin (VA), teicoplanin (TEC)), oxazolidinones class (linezolid (LZD), tedizolid (TZD), and contezolid (CZD)], lipoglycopeptides class (dalbavancin (DAL), oritavancin (ORI)) and cyclic lipopeptides (daptomycin (DAP)) have difficulty entering into the OM of GNB and binding to their intracellular target sites. Most antibacterial targets are highly conserved across Gram-positive bacteria and GNB, and a lack of compound entry has been demonstrated by the GNB intracellular accumulation analysis by liquid chromatography with tandem mass spectroscopy (LC–MS/MS) [10]. In addition, several studies have shown that GNB with permeabilized outer membranes or impaired efflux pumps are vulnerable to GPOAs [11, 12]. This makes it reasonable to assume that the permeabilizing properties of polymyxins could help GPOAs to penetrate the OM of GNB. However, variations in lipopolysaccharide structure, porins and efflux pumps of different GNBs affect the effects of polymyxins on GPOAs; in this regard, it is still unclear which GPOAs that polymyxins might better aid in penetrating which type of GNB.

To solve the above problem, in this study, we assessed the in vitro synergistic activity of combinations of polymyxins and GPOAs against the following GNBs noted on the WHO’s priority list: *Acinetobacter baumannii* (*A. baumannii*), *Escherichia coli (E. coli)*, *Klebsiella pneumoniae* (*K. pneumoniae*), and *Pseudomonas aeruginosa* (*P. aeruginosa*).

## Materials and methods

### Antibiotics

COL, PMB, VA, LZD, TZD and DAP were purchased from Bidepharm (Shanghai, China), DAL and ORI were obtained from Shanghai Minbiotech Co., Ltd (Shanghai, China), TEC was purchased from Shanghai Macklin Biochemical Co., Ltd (Shanghai, China), CZD was obtained from Shanghai MicuRx Pharmaceutical Co., Ltd (Shanghai, China). Stock solutions of 2560 μg/mL (COL and PMB) and 5120 μg/mL (VA, LZD, TEC, and DAP) were prepared in sterile distilled water, and 51,200 μg/mL (TZD, CZD, and DAL) were dissolved in dimethyl sulfoxide (DMSO). ORI susceptibility assays included 0.002% polysorbate 80 to minimize the loss of ORI to plastic surfaces [13].

### Bacterial isolates

Four ATCC reference strains (*A. baumannii* ATCC 19606, *P. aeruginosa* ATCC 27853, *K. pneumoniae* ATCC 700603, and *E. coli* ATCC 25922) were obtained from the department of laboratory medicine, General Hospital of Southern Theatre Command. Nine clinical isolates (*A. baumannii* (316039, 214024 and 235875), *P. aeruginosa* (304238 and 120014), *K. pneumoniae* (327004, 325016, 418015 and 235094), and *E. coli* (103231 and 222061)) used in this study were identified by Vitek MS system (bioMérieux, Marcy-l'Étoile, France). Antimicrobial susceptibility testing of all clinical isolates was performed by Vitek-2 compact (bioMérieux, Marcy- l'Étoile, France). The quality control stains used were *E. coli* ATCC 25922 and *P. aeruginosa* ATCC 27853.

### Minimal inhibitory concentration (MIC) assays

The MICs of polymyxins and GPOAs were determined in triplicate using the broth microdilution method and according to the Clinical and Laboratory Standards Institute (CLSI) 2020 guidelines [14]. All experiments were conducted using freshly prepared Cation-Adjusted Mueller–Hinton Broth (CAMHB), which was purchased from Qingdao hopebiol Biotechnology Co., Ltd (Shandong, China). 100 μL of inoculum was adjusted to roughly 5 × 10^5^ CFU/mL, and the microtiter plates were visually observed after incubation for 16–20 h at 37 ℃. The breakpoints were interpreted according to CLSI 2020.

### Checkerboard assays

The synergy test of polymyxins with GPOAs was performed by standard checkerboard method using 96-well microtiter plates. Each antibiotic was prepared by doubling dilutions of GPOAs (0–512 μg/mL) in the vertical wells and polymyxins (0–16 μg/mL) in the horizontal wells. The final concentration of bacterial suspension was adjusted to approximately 5 × 10^5^ CFU/mL in a 100 μL final volume. The plates were incubated at 37 ℃ for 16–20 h. Assays were performed in triplicate for each isolate. The fractional inhibitory concentration index (FICI) was calculated for each combination with the following equation: FICI = FIC_A_ + FIC_B_, where FIC_A_ = MIC of drug A in combination/MIC of drug A alone, and FIC_B_ = MIC of drug B in combination/MIC of drug B alone. The interpretation of the FICI was as follows: FICI ≤ 0.5, synergism; FICI > 0.5 and ≤ 4, indifference; FICI > 4, antagonism [15].

### Time-kill assays

This experiment was conducted in triplicate to evaluate the bactericidal activity of both agents and their combinations. Based on the MIC results, a final concentration of either 0.5 or 1 µg/mL (1 × MIC) was chosen for COL. DAL and ORI were both added at 20 µg/mL to achieve concentrations in plasma [16]. 100 µL of the 0.5 McFarland GNB suspension was added to 5 mL of CAMHB at 37℃ until it reached log-phase growth (approximately 4 h). The culture was then adjusted to the turbidity of a 1.0 McFarland in CAMHB, and 100 µL was added to each of the antimicrobial solutions. Tubes without antibiotics were used as growth controls. Colony counts were observed at 0, 2, 4, 8, 18 and 24 h. A tenfold dilution series was prepared in CAMHB and a 10 μL drop of each dilution was transferred to Mueller–Hinton plates and incubated overnight at 37 °C in ambient air for 16–20 h. Bactericidal activity was defined as a ≥ 3 log_10_ reduction in colony count compared with the bacterial concentration of starting inoculum. Synergy was defined as a ≥ 2 log_10_ decrease in colony count compared to the effect of the most active single agent after 24 h [17].

## Results

### Antimicrobial susceptibility and synergy testing by checkerboard assays

As shown in Table S1, four of these strains were MDR isolates, six were XDR isolates and two were PDR isolates. All strains were susceptible to COL except *P. aeruginosa* 235875 and *K. pneumoniae* 418015 and 235094. Regarding β-lactam antibiotics such as cephalosporins, carbapenems and monobactam, all clinical strains exhibited nearly complete resistance. For aminoglycosides, four strains of *K. pneumoniae* demonstrated resistance, whereas the other GNB strains exhibited a mix of sensitive and resistant strains. All strains displayed either a resistant or intermediate phenotype when they were exposed to fluoroquinolones. Sensitive strains were predominated for the tetracyclines, particularly for tigecycline. Conversely, almost all strains exhibited resistance to sulfonamides.

In the checkerboard assay, all GPOAs alone had MICs not less than 128 μg/mL for all four ATCC strains and nine clinical strains. With the exception of *P. aeruginosa* 235875, *K. pneumoniae* 418015 and 235094, all strains exhibited MICs ranging from 0.25 μg/mL to 2 μg/mL when treated solely with polymyxins. In the combination tests, we focus not only on combinations with FICI ≤ 0.5, but also on combinations where polymyxins at 0.5 × MIC can reduce GPOAs to no more than 32 μg/mL (steady state plasma concentration achievable by some GPOAs), the latter of which we call mild synergy. As demonstrated in Table 1, no antagonisms were observed. Among a total of 15 strains, the combinations of COL-DAL, COL-ORI, COL-VA, COL-LZD, COL-TZD, COL-TEC, COL-CZD, and COL-DAP had synergistic effects on 14 (93.3%), 11 (73.3%), 9 (60.0%), 8 (53.3%), 4 (26.7%), 3 (20.0%), 2 (13.3%) and 1 (6.7%) strains, respectively. Compared to COL-based combinations, PMB-based combinations of PMB-ORI, PMB-VA, PMB-LZD, PMB-DAL, PMB-TEC, PMB-DAP, PMB-TZD and PMB-CZD exhibited synergistic effects in 10 (66.7%), 9 (60.0%), 7 (46.7%), 5 (33.3%), 5 (33.3%), 3 (20.0%), 3 (20.0%) and 1 (6.7%) strains, respectively. In general, combinations based on COL are slightly better than those based on PMB. ORI, DAL, VA, LZD and TEC show better results when used in combination with polymyxins compared to TZD, CZD and DAP.

**Table 1:** FICI values of polymyxins combined with GPOAs against fifteen isolates

		Synergy testing results							
		Lowest FICI							
Bacteria	Strain	COL-DAL	COL-ORI	COL-VA	COL-LZD	COL-TEC	COL-TZD	COL-CZD	COL-DAP
*A. baumannii*	ATCC19606	**0.31**	**0.27**	***0.53***	**0.50**	**0.51**	**0.50**	1.50	1.50
*A. baumannii*	316039	**0.31**	**0.28**	**0.38**	0.75	**0.50**	1.00	0.75	1.50
*A. baumannii*	214024	**0.38**	**0.13**	**0.28**	***0.52***	***0.63***	**0.52**	1.00	***0.63***
*A. baumannii*	235875	**0.13**	**0.13**	**0.16**	0.63	**0.19**	**0.53**	1.00	0.75
*P. aeruginosa*	ATCC27853	1.50	**0.26**	**0.50**	**0.50**	***0.53***	**0.50**	1.50	1.50
*P. aeruginosa*	304238	**0.31**	***0.51***	1.00	1.50	***0.63***	1.00	***0.63***	1.50
*P. aeruginosa*	120014	**0.50**	***0.63***	***0.53***	**0.50**	***0.52***	**0.63**	**0.50**	0.75
*E. coli*	ATCC25922	**0.28**	**0.26**	**0.25**	0.75	1.50	**0.50**	1.50	1.50
*E. coli*	103231	**0.50**	1.50	**0.31**	**0.25**	***0.63***	**0.25**	0.75	***0.56***
*E. coli*	222061	**0.19**	**0.16**	**0.27**	***0.56***	***0.63***	1.00	***0.53***	1.00
*K. pneumoniae*	ATCC700603	**0.50**	**0.27**	**0.50**	1.50	0.63	1.50	1.50	1.50
*K. pneumoniae*	327004	**0.50**	**0.26**	1.00	**0.50**	1.50	1.50	***0.63***	1.50
*K. pneumoniae*	325016	**0.38**	**0.14**	***0.51***	**0.38**	**0.50**	1.50	1.50	**0.50**
*K. pneumoniae*	418015	**0.50**	***0.56***	**0.50**	**0.09**	0.75	0.75	**0.13**	0.75
*K. pneumoniae*	235094	**0.38**	**0.25**	0.75	**0.13**	1.00	1.00	***0.63***	1.00

Bacteria	Strain	PMB-DAL	PMB-ORI	PMB-VA	PMB-LZD	PMB-TEC	PMB-TZD	PMB-CZD	PMB-DAP
*A. baumannii*	ATCC19606	**0.27**	**0.27**	0.75	1.00	**0.38**	0.63	1.00	**0.50**
*A. baumannii*	316039	**0.25**	**0.16**	**0.28**	**0.50**	**0.28**	1.00	1.00	**0.50**
*A. baumannii*	214024	0.75	1.00	**0.16**	***0.63***	**0.31**	1.00	1.00	0.75
*A. baumannii*	235875	**0.25**	**0.38**	**0.50**	0.75	**0.28**	1.00	1.00	0.75
*P. aeruginosa*	ATCC27853	1.50	**0.28**	1.50	1.50	1.50	1.50	1.50	1.50
*P. aeruginosa*	304238	1.50	***0.52***	***0.63***	0.75	1.50	1.00	1.50	1.50
*P. aeruginosa*	120014	***0.52***	**0.38**	***0.63***	0.63	0.75	1.50	0.75	**0.50**
*E. coli*	ATCC25922	***0.53***	1.50	**0.50**	0.75	***0.51***	1.50	1.50	***0.52***
*E. coli*	103231	0.75	**0.50**	**0.38**	**0.50**	0.75	**0.50**	1.00	1.00
*E. coli*	222061	**0.31**	**0.13**	**0.26**	**0.25**	**0.25**	1.50	1.50	***0.63***
*K. pneumoniae*	ATCC700603	***0.52***	**0.14**	**0.28**	**0.38**	1.50	**0.50**	1.50	1.50
*K. pneumoniae*	327004	**0.38**	**0.28**	**0.50**	**0.38**	***0.53***	1.50	1.00	1.50
*K. pneumoniae*	325016	***0.51***	**0.31**	1.50	1.50	***0.56***	**0.50**	1.00	1.50
*K. pneumoniae*	418015	***0.56***	***0.53***	**0.50**	**0.19**	0.75	0.75	**0.38**	***0.63***
*K. pneumoniae*	235094	1.00	1.00	***0.63***	**0.13**	1.00	1.00	***0.53***	1.00

The black bold numbers indicate a synergistic effect, while the bold italic numbers indicate a mild synergistic effect

VA: vancomycin; TEC: teicoplanin; LZD: linezolid; TZD: tedizolid; CZD: contezolid; DAL: dalbavancin; ORI: oritavancin; DAP: daptomycin; COL:colistin; PMB: polymyxin B

### Time-kill assays

**Fig. 1:**
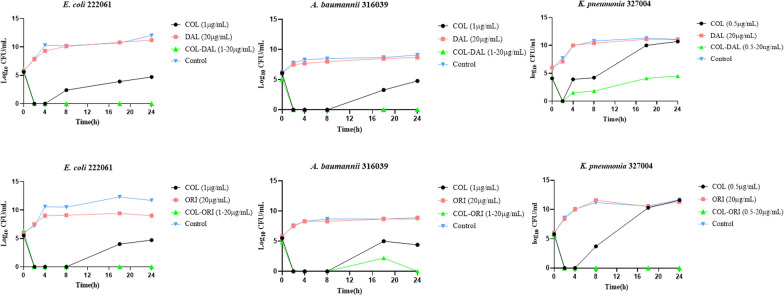
Time-kill studies of colistin alone and in combination with dalbavancin and oritavancin. Data were obtained from clinical isolates of *A. baumannii, E. coli and K. pneumoniae*

Using time–kill methodology with colistin-susceptible isolates, Fig. 1 shows the synergistic effect of COL in combination with DAL or ORI against three strains (*E. coli* 222061, *A. baumannii* 316039, and *K. pneumoniae* 327004). Exposure to DAL or ORI alone had no inhibitory effect on bacterial growth, although COL alone at 1 × MIC was initially bactericidal against all strains tested, there was rapid regrowth after a maximum of 8 h. The combination of DAL or ORI with COL also resulted in rapid bactericidal effects, but regrowth did not occur even after 24 h of incubation, except for *K. pneumoniae* 327004 treated with DAL and COL.

## Discussion

The global spread of drug-resistant GNB, such as carbapenemase-producing *Enterobacteriaceae* and *A. baumannii*, has prompted recurrent requests for research and development (R&D) of new antibiotics. Despite the urgency for new antibiotics, the global antibiotic pipeline is meagre. Recognizing this crisis, a review of previously unpopular antibiotics and unconventional combinations are certainly necessary and feasible.

The ability of polymyxins to disturb outer membrane permeability has been previously used to investigate the potential for synergy of colistin in combination with GPOAs, such as VA [18], DAP [19], LZD [17, 20] and TEC [21]. However, most of these studies have only investigated the synergistic effect of a GPOA in combination with polymyxins on one kind of bacteria, and polymyxins have largely chosen colistin rather than polymyxin B. In order to reduce the nephrotoxicity of polymyxins by reducing their dose and to broaden the antibacterial spectrum of GPOAs, a systematic study is urgently needed to compare the synergistic effect of all GPOAs in combination with polymyxins (colistin and polymyxin B) on four kinds of clinically common GNB with severe antibiotic resistance.

From the perspective of each bacterial strain, when considering the spectrum of synergistic effects for different polymyxins/GPOAs combinations, we can draw the following conclusions: for strains belonging to the same type of bacteria, the differences in the spectrum of synergistic efficacy between the reference strain (wild type drug-sensitive strain) and clinical drug-resistant strain are not greater than those between clinical drug-resistant strains; the synergistic effects on *P. aeruginosa* strains are significantly lower than those on strains of *A. baumannii*, *K. pneumoniae* and *E.coli*; as to *K. pneumoniae*, it appears that its resistance to polymyxins does not affect the synergistic effects exhibited by specific polymyxins/GPOAs combinations.

Although the synergies between GPOAs and polymyxins was still species and strain specific, these data suggest that the combination of GPOAs-polymyxins could be a useful option for the treatment of complicated GNB infections. Considering GPOAs have potent and rapidly bactericidal activity against Gram-positive bacteria, which may be advantageous when mixed Gram-positive and Gram-negative infections are presented or suspected. As some of GPOAs administration alone is also associated with significant nephrotoxicity, there may be concerns over its use in combination with polymyxins. Nevertheless, it should be noticed that the concentrations of polymyxins required to mediate GPOAs synergy in vitro are relatively low, which may reduce the risk of developing renal impairment if the antibiotics are given together. As reported in the literature [18], Wareham et al. have identified a number of patients with MDR *A. baumannii* who received colistin with concomitant vancomycin therapy for the treatment of Gram-positive bacteria-associated infections without apparent nephrotoxicity. Concerns over GPOAs-polymyxins renal impairment may also be less important if polymyxins were to be administered in aerosolized form. Nebulized polymyxins have been increasingly used in the treatment of GNB respiratory tract infections in several diseases (e.g., ventilator-associated pneumonia and chronic cystic fibrosis infections), but there is little evidence to demonstrate that they cause nephrotoxicity when delivered via a nebulizer.

We were unable to demonstrate synergy versus polymyxins resistant isolate in the time-kill study (data not shown), most likely due to the inability of polymyxins to disrupt the OM with rapidly changing components of the strain. Of particular interest in the checkerboard assay, synergy and bactericidal activities were demonstrated for COL/VA, COL/CZD, PMB/VA and PMB/CZD at 0.25 × or 0.5 × MIC against *K. pneumoniae* 418015, but not against colistin susceptible isolates of *K. pneumoniae*. As observed and previously suggested by Duval et al. [22], the presence of higher proportions of heterogeneous subpopulations resistant to colistin in *K. pneumoniae* 418015 may favor the OM penetration and activity of the unconventional antibiotics tested.

Our results demonstrated that the synergistic spectrum of polymyxins-GPOAs is species and strain specific. As previously published by Zgurskaya et al. [23], the presence of the OM and a plethora of active efflux pumps determine the amount of antibiotic accumulation in the GNB, which is positively correlated with the activity of the antibiotic. Different GNB pathogens differ dramatically in their permeability barriers, with the OM playing the dominant role in *E. coli* and *P. aeruginosa* but efflux pump dominating in *A. baumannii*, which results in huge differences in the accumulation of compounds with different chemical structures and even of the same chemotype in GNB. Consistent with the conclusion that in *P. aeruginosa*, all antibiotics were impacted by both the OM and efflux, while in *A. baumannii* and *E. coli*, the contributions of the OM and efflux were antibiotic specific. We also found that the synergistic interactions on *P. aeruginosa* strains are significantly lower than those on strains of *A. baumannii*, *K. pneumoniae* and *E. coli*. Polymyxins disrupt the OM structure of GNB and the efflux pump in it, thereby facilitating the entry of GPOAs into GNB through different permeation pathways and reducing efflux to exert different antibacterial effects.

Although our data suggest and somewhat confirm that polymyxins-GPOAs combinations might result in synergistic and bactericidal activity, but this remains to be confirmed. A pharmacokinetic study will be needed to assess how best to dose and monitor the two antibiotics in combination before any firm conclusions can be drawn. Further investigations of polymyxin-GPOAs combinations in animal infection models and patients are warranted to optimize the target species and strains and administration dosage. A successful application of polymyxin-GPOAs combination in the clinic requires collaboration between clinical microbiologists and clinicians, starting with rapid in vitro testing (Etest method would be an appropriate option) of the antibacterial efficacy of the combination on the strains from the patient by clinical microbiologist, followed by clinician selection of the appropriate combination and dose based on in vitro susceptibility results and in vivo data.

## Conclusion

In summary, we have provided in vitro evidence that polymyxin-GPOAs combinations have potential synergistic efficacy against four GNB pathogens of high clinical importance. With very few new antibiotics likely to become available for the treatment of severe resistant GNB in the next 5 to 10 years, polymyxin-GPOAs combinations could be considered potential therapies that can help bridge the R&D gap. Further in vivo and clinical studies will be needed to support our results.

### Supplementary Information

Supplementary Material 1.

## Data Availability

Data is provided within the manuscript or supplementary information files.
