# Supplementary material for: Potent synergy and sustained bactericidal activity of polymyxins combined with Gram-positive only class of antibiotics versus four Gram-negative bacteria
Source: Ann Clin Microbiol Antimicrob. 2024 Jul 4;23:60. doi: 10.1186/s12941-024-00720-4 (PMC11225234; doi:10.1186/s12941-024-00720-4)
Supplement: Supplementary file 1 — Supplementary Material 1. [file 12941_2024_720_MOESM1_ESM.docx]

**Potent Synergy and Sustained Bactericidal Activity of Polymyxins Combined with Gram-Positive Only Class of Antibiotics Versus Three Gram-Negative Bacteria**

Yan Wang^1,2^, Jianwen Feng^1,2^, Jiameng Yu^3^, Lirong Wen^4^, Lidan Chen^5^, Huijie An^2^, Weibin Xiao^6^, Bing Zhang^7^, Huanhuan Feng^7^, Mou Zhou^8^, Zhihui Jiang^1,2,3*^

^1^School of pharmaceutical sciences, Southern Medical University, Guangzhou 510515, China.

^2^Department of Pharmacy, General Hospital of Southern Theatre Command, Guangzhou 510010, China.

^3^Graduate School, Guangzhou University of Chinese Medicine, Guangzhou 510006, China.

^4^School of Pharmaceutical Sciences, Dali University, Dali 671003, China

^5^Department of Laboratory Medicine, General Hospital of Southern Theatre Command, Guangzhou 510010, China.

^6^Department of Clinical Pharmacy, General Hospital of Southern Theatre Command, Guangzhou 510010, China.

^7^Department of Healthcare, General Hospital of Southern Theatre Command, Guangzhou 510010, China.

^8^Department of Blood Transfusion Medicine, General Hospital of Southern Theatre Command, Guangzhou 510010, China.

*Corresponding author.

E-mail address: [jandsphy@163.com](mailto:jandsphy@163.com)

**Table of contents**

**1 Table S1.** MIC (mg/L) and susceptibility profiles of eleven clinical isolates.

**Table S1** MIC (mg/L) and susceptibility profiles of eleven clinical isolates

|  | *A. baumannii* | | | *P. aeruginosa* | | *E. coli* | | *K. pneumoniae* | | | |
| --- | --- | --- | --- | --- | --- | --- | --- | --- | --- | --- | --- |
|  | 316039 | 214024 | 235875 | 304238 | 120014 | 103231 | 222061 | 327004 | 325016 | 418015 | 235094 |
| Antibiotics | MIC (susceptibility profile) | | | | | | | | | | |
| Colistin | 0.5 (S) | 0.5 (S) | 16 (R) | 0.5 (S) | 0.5 (S) | 0.5 (S) | 0.5 (S) | 0.5 (S) | 0.5 (S) | 16 (R) | 8 (R) |
| Tigecycline | 2 (S) | 1 (S) | ND | ND | ND | 0.5 (S) | 0.5 (S) | 0.5 (S) | 8 (R) | 2 (R) | 4 (I) |
| Cefepime | 32 (R) | 32 (R) | 32 (R) | 16 (I) | 32 (R) | 32 (R) | 8 (SDD) | 32 (R) | 32 (R) | 32 (R) | 16 (I) |
| Cefoperazone/Sulbactam | 64 (R) | 64 (R) | 64 (R) | 64 (R) | 64 (R) | 64 (R) | 64 (R) | 64 (R) | 64 (R) | 64 (R) | 32 (R) |
| Piperacillin/Tazobactam | 128 (R) | 128 (R) | 128 (R) | ND | ND | 128 (R) | 128 (R) | 128 (R) | 128 (R) | 128 (R) | 128 (R) |
| Ticarcillin/Clavulanic acid | 128 (R) | 128 (R) | 128 (R) | 128 (R) | 128 (R) | 32 (R)^a^ | 32 (R)^a^ | 128 (R) | 128 (R) | 128 (R) | 128 (R) |
| Ceftazidime | 64 (R) | 64 (R) | 64 (R) | 32 (R) | 64 (R) | 32 (R) | 64 (R) | 64 (R) | 64 (R) | 64 (R) | 64 (R) |
| Imipenem | 16 (R) | 16 (R) | 16 (R) | 8 (R) | 16 (R) | 0.25 (S) | 16 (R) | 16 (R) | 16 (R) | 16 (R) | 16 (R) |
| Meropenem | 16 (R) | 16 (R) | 16 (R) | 4 (I) | 8 (R) | ND | ND | 16 (R) | 16 (R) | 16 (R) | 16 (R) |
| Aztreonam | ND | ND | 64 (R) | ND | ND | ND | ND | 64 (R) | 64 (R) | 64 (R) | ND |
| Amikacin | ND | ND | 64 (R) | 2 (S) | 64 (R) | 16 (S) | 2 (S) | 64 (R) | 64 (R) | 64 (R) | ND |
| Tobramycin | 1 (S) | 16 (R) | 16 (R) | 1 (S) | 16 (R) | ND | ND | 16 (R) | 16 (R) | 16 (R) | 16 (R) |
| Ciprofloxacin | 4 (R) | 4 (R) | 4 (R) | 1 (I) | 4 (R) | ND | ND | 4 (R) | 4 (R) | 4 (R) | 4 (R) |
| Levofloxacin | 4 (I) | 8 (R) | 8 (R) | 4 (R) | 8 (R) | 8 (R) | 8 (R) | 8 (R) | 8 (R) | 8 (R) | 8 (R) |
| Minocycline | 4 (S) | 4 (S) | 16 (R) | ND | ND | ND | ND | 4 (S) | 16 (R) | 16 (R) | 16 (R) |
| Trimethoprim-Sulfamethoxazole | 8/152 (R) | 16/304 (R) | 16/304 (R) | 16/304 (R) | ND | 16/304 (R) | 16/304 (R) | 16/304 (R) | 1/19 (S) | 16/304 (R) | 16/304 (R) |

R, resistance; S, sensitivity; I, intermediate. SDD, susceptible-dose dependent. -, intrinsic resistance. ND, not determined. ^a^: Amoxicillin/Clavulanic acid
